# Light affects tissue patterning of the hypocotyl in the shade-avoidance response

**DOI:** 10.1371/journal.pgen.1008678

**Published:** 2020-03-23

**Authors:** Esther Botterweg-Paredes, Anko Blaakmeer, Shin-Young Hong, Bin Sun, Lorenzo Mineri, Valdeko Kruusvee, Yakun Xie, Daniel Straub, Delphine Ménard, Edouard Pesquet, Stephan Wenkel

**Affiliations:** 1 Copenhagen Plant Science Centre, University of Copenhagen, Thorvaldsensvej, Denmark; 2 Department of Plant and Environmental Sciences, Faculty of Science, University of Copenhagen, Copenhagen, Denmark; 3 Department of Biosciences, University of Milan, Milan, Italy; 4 Centre for Plant Molecular Biology (ZMBP), University of Tübingen, Germany; 5 Quantitative Biology Center (QBiC), University of Tübingen, Auf der Morgenstelle, Tübingen, Germany; 6 Microbial Ecology, Center for Applied Geoscience, University of Tübingen, Tübingen, Germany; 7 Arrhenius Laboratories, Department of Ecology, Environment and Plant Sciences (DEEP), Stockholm University, Stockholm, Sweden; 8 NovoCrops Center, University of Copenhagen, Thorvaldsensvej, Denmark; Peking University, CHINA

## Abstract

Plants have evolved strategies to avoid shade and optimize the capture of sunlight. While some species are tolerant to shade, plants such as *Arabidopsis thaliana* are shade-intolerant and induce elongation of their hypocotyl to outcompete neighboring plants. We report the identification of a developmental module acting downstream of shade perception controlling vascular patterning. We show that Arabidopsis plants react to shade by increasing the number and types of water-conducting tracheary elements in the vascular cylinder to maintain vascular density constant. Mutations in genes affecting vascular patterning impair the production of additional xylem and also show defects in the shade-induced hypocotyl elongation response. Comparative analysis of the shade-induced transcriptomes revealed differences between wild type and vascular patterning mutants and it appears that the latter mutants fail to induce sets of genes encoding biosynthetic and cell wall modifying enzymes. Our results thus set the stage for a deeper understanding of how growth and patterning are coordinated in a dynamic environment.

## Author summary

Shade sensitive plants such as Arabidopsis respond to shade by growing tall in order to maximize their access to sunlight. We find that the REVOLUTA (REV) and KANADI1 (KAN1) transcription factors which are primarily involved in patterning the early leaf, impinge on the regulation of *WUSCHEL HOMEOBOX4* (*WOX4*), another transcription factor involved in vascular development. The regulation of *WOX4* leads to an increase of the number of water-conducting xylem cells in response to shade. Consequently, mutations in the genes encoding either REV, KAN1 or WOX4 are impaired in their ability to grow tall in shade. Thus, we have uncovered a connection between basic patterning and adaptive growth.

## Introduction

Shade intolerant plants such as Arabidopsis respond to subtle changes in the red (R) to far-red (FR) light ratio (R:FR) by increasing hypocotyl elongation growth to outcompete shade caused by neighboring plants. When shaded, several key transcription factors change rapidly to induce transcriptional signaling cascade(s) impinging on genes encoding components of auxin production and signaling [1–4]. This rapid response relies on the activity of the phytochrome photoreceptor system in which PHYTOCHROME-INTERACTING FACTORs (PIFs) form the first layer downstream of PHYTOCHROME B (PHYB). In white light conditions, when the R:FR ratio is high, PHYB is active in the nucleus where it maintains the continuous degradation of PIF transcription factors [5]. In shade, the R:FR ratio is low, which inactivates PHYB and thus increases the half-life of PIFs, mainly PIF3, PIF4 and PIF7, to activate genes encoding auxin biosynthesis enzymes [6]. Among the auxin biosynthesis enzymes required for a full shade avoidance response, TRYPTOPHAN AMINOTRANSFERASE OF ARABIDOPSIS1 (TAA1) and different members of the YUCCA family produce auxin directly from tryptophan in a shade-responsive manner [7–9]. In addition to PIFs, genes encoding class II homeodomain leucine zipper (HD-ZIPII) transcription factors, especially *HAT1*, *HAT2*, *HAT3*, *HAT4*/*ATHB2* and *ATHB4*, are also rapidly induced in response to a low R:FR ratio [2, 10–12] partially mediated by PIFs [13, 14]. These HD-ZIPII transcription factors have an N-terminal EAR motif used to form transcriptional repressor complexes with TOPLESS/TOPLESS-related co-repressors [15]. In fact, ATHB4 acts as a transcriptional repressor on targets such as the *YUCCA5* gene encoding an auxin biosynthetic enzyme [16]. The complex regulation of *YUCCA5* supports the idea that HD-ZIPIIs impede auxin production to dampen PIF function and avoid excessive auxin-dependent growth. At the initiation of the shade-induced elongation growth, recent genetic studies revealed a decisive role of the hypocotyl epidermis in the production and dissipation of auxin signaling [17]. It however remains unknown whether the different hypocotyl cell-types divide, elongate and/or differentiate to enable the shade-dependent growth. Genome-wide transcription factor binding site studies focusing on the master patterning factors of the class III homeodomain leucine zipper (HD-ZIPIII) as well as on KANADI families revealed several direct target genes with known roles in the shade avoidance response [18–20]. Interestingly, these HD-ZIPIII proteins also interact with the rapid shade-response HD-ZIPII transcription factors to regulate leaf patterning [21–23].

Here we show that the HD-ZIPIII transcription factor REVOLUTA (REV) and the KANADI transcriptional repressor KANADI1 (KAN1) impinge on the transcriptional regulation of the WUSCHEL RELATED HOMEOBOX 4 (WOX4) transcription factor that acts as a master regulator of (pro)cambium maintenance. In response to shade, Arabidopsis seedlings induce hypocotyl growth (extended elongation and limited girth increase) which is accompanied by an increase in tracheary elements (TEs), these xylem cells provide both axial mechanical support as well as function in hydro-mineral sap conduction. Both numbers and types of TEs changed with shading, inducing the formation of more metaxylem-type TEs characterized by larger luminal diameter and reticulate/pitted cell wall organization. Plants with mutations in either *REV*, *KAN1* or *WOX4* showed defects in hypocotyl elongation and were unable to increase the number of TEs in the vascular cylinder. Moreover, the xylem patterning response that we have observed extends beyond Arabidopsis and together with additional *in vitro* data, we have uncovered a transcriptional circuitry that controls environment-sensitive cell fate transitions governing plant plasticity to respond to shade.

## Results

### *WOX4* is a direct REVOLUTA and KANADI1 target gene

**Fig 1:**
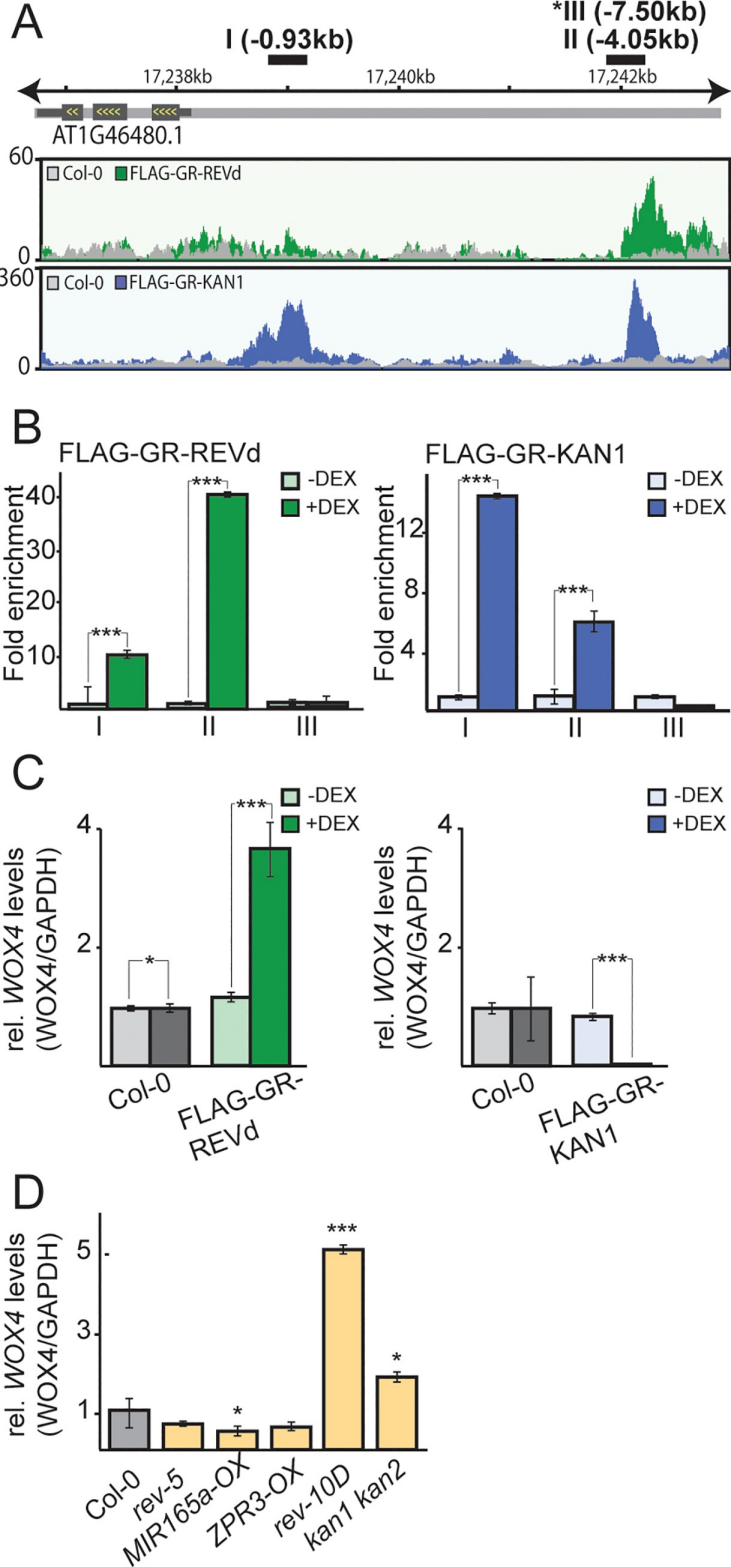
*WOX4* is a direct target gene of the HD-ZIPIII transcription factor REVOLUTA and the GARP-transcription factor KANADI1. **A**, Organization of the *WOX4* locus. Fragments I-III indicate the positions tested by ChIP-qPCR. Plotted below the gene model are the read coverages obtained from ChIP-seq experiments. Asterisk indicates that primer pair III is not present in the read coverage plots because it is located outside the depicted region. **B**, ChIP-qPCR experiments with transgenic *35S*::*FLAG-GR-REVd* and *35S*::*FLAG-GR-KAN1* plants that were either mock-treated (-DEX, light green/blue bars) or treated with dexamethasone (+DEX, dark green/blue bars). The genomic regions were tested with three primer pairs (I–III). The y axis shows the fold enrichment normalized to the mock-treated immunoprecipitations. **C**, Real-time quantitative reverse transcription (RT)-PCR experiments that show expression changes of *WOX4* in Col-0 (grey), *35S*::*FLAG-GR-REVd* (green) and *35S*::*FLAG-GR-KAN1* (blue) in response to either 60 min. (*REV*) or 120 min. (*KAN1*) of DEX induction. Average expression levels of three biological replicates are plotted, normalized to GAPDH of the ratio +DEX versus–DEX treatments with standard error. **D**, Real-time quantitative reverse transcription (RT)-PCR experiments that show expression changes of *WOX4* in Col-0 and plants with altered *HD-ZIPIII* or *KANADI* expression. Average expression levels of three biological replicates are plotted, normalized to GAPDH with standard error. T-Tests *p≤ 0.05, ***p≤0.001.

Using ChIP-seq, we previously identified genome-wide binding sites for the HD-ZIPIII transcription factor REVOLUTA (REV) and the GARP-type transcription factor KANADI1 (KAN1) [18, 19]. Both REV and KAN1 antagonistically regulate patterning of early leaf primordia: in this context REV determines the adaxial (future upper) and KAN1 the abaxial (future lower) side of the developing leaf. Comparative studies combining ChIP- and mRNA-sequencing as well as microarray-based technologies revealed a number of targets directly regulated REV and KAN1 [18–20, 24, 25], several of which have known roles in the shade avoidance response. The transcription factor *WUSCHEL-RELATED HOMEOBOX4* (*WOX4*) was among the genes that are directly and oppositely regulated by REV and KAN1. Our ChIP-seq data showed two binding peaks for KAN1 (-1.0kb and -4.0kb) upstream of the transcription start site while REV showed only one peak (-4.0kb upstream) indicating that REV and KAN1 could compete for chromatin access at the more distant position (Fig 1A). We verified the binding of REV and KAN1 to these respective positions by performing independent ChIP-qPCRs and tested a region further upstream as a negative control (Fig 1B). Transgenic plants expressing fusions of REV or KAN1 to the glucocorticoid receptor exhibited robust changes of *WOX4* mRNA levels when treated with dexamethasone: REV induced while KAN1 repressed *WOX4* expression (Fig 1C). This confirmed that REV acts predominantly as a transcriptional activator and KAN1 as a transcriptional repressor [18–20, 24, 25]. Finally, we assessed the levels of *WOX4* mRNA in different *rev* and *kan* mutants: plants with reduced *REV* or *HD-ZIPIII* levels such *MIR165a-OX*, exhibited significantly lower levels of *WOX4* mRNA whereas plants with either a *REV* gain-of-function mutation (*rev10D*) or mutated in *KAN1* and *KAN2* (*kan1 kan2*) had significantly increased levels of *WOX4* mRNA (Fig 1D). Taken together, these findings support a role for REV and KAN1 as direct upstream regulators of *WOX4*.

Early preliminary studies revealed a reduction of the procambial layer in the vascular bundles of petioles when plants were grown in shade (S1 Fig), leading us to investigate the relationship between vascular patterning and shade growth. WOX4 is an important regulator of (pro)cambial identity in the plant vasculature [26–28]. (Pro)cambium cells have stem cell properties and can differentiate into tracheary elements, which both allow mechanical support and water-conduction, and are essential to form wood in perennial plants. Loss of *WOX4* causes a strong reduction of cambium formation in *Arabidopsis* [28] and a strong reduction in girth of the main stem in poplar [29].

Using histological analysis of the stem bases, we investigated if *REV* and *WOX4* genetically interacted to regulate (pro)cambium development in Col-0 wild type, *35S*::*WOX4*, *rev5*, *wox4* and *rev5 wox4* mutant plants (S2 Fig). Compared to Col-0 wild type plants, no significant vascular changes were observed in transgenic *35S*::*WOX4* plants (S2 Fig) whereas *wox4* mutant plants displayed, as previously reported, a significant reduction in the amount of cambium/forming xylem (S2 Fig). In contrast, *rev5* mutants showed deformed, flattened vascular bundles but no visible reduction in cambium (S2 Fig). The *wox4 rev5* double mutant cumulatively showed both reduced cambium as well as deformed and flattened vascular bundles together with an increase in tissue surrounding the vasculature (S2 Fig). Thus, it seems, that the latter effects are additive and do not point towards a genetic interaction of the REV and WOX4 pathways in controlling cambium maintenance in the main stem. In summary, these observations suggest that *WOX4* expression is regulated by REV and KAN1 but this regulation is not important for the vascular patterning of the main stem.

### Loss of *WOX4* function affects the shade avoidance response

**Fig 2:**
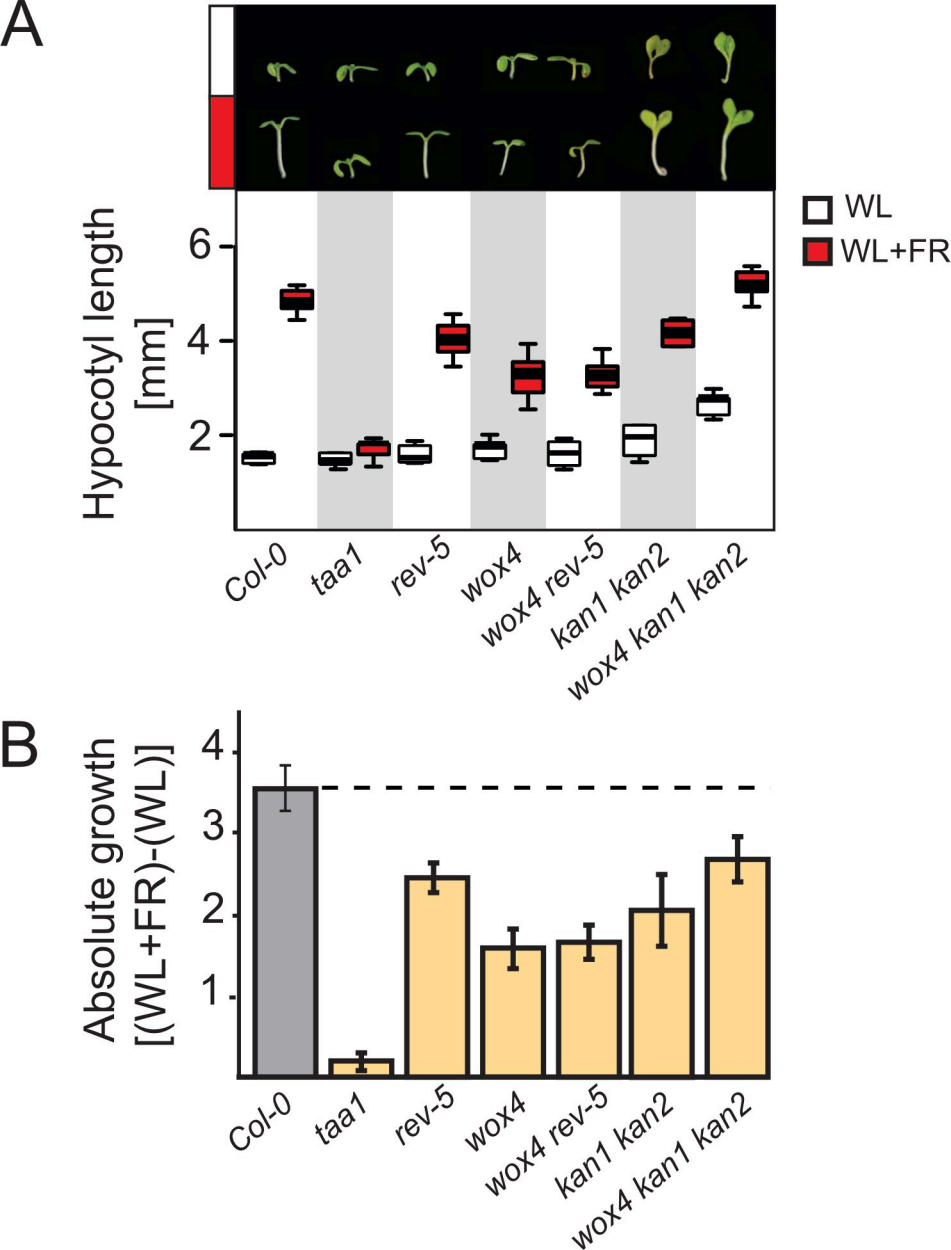
Mutants affecting vascular patterning show altered shade avoidance hypocotyl responses. **A**, Hypocotyl lengths of wild type Col-0 and an array of mutant seedlings in white light (WL) and far-red enriched white light (WL+FR) conditions. The upper panel depicts representative seedlings. Box plots below show the observed experimental data; white boxes hypocotyls grown in WL; red boxes, hypocotyls grown in WL+FR. **B**, Absolute hypocotyl expansion by subtracting the length in WL from the length in WL+FR.

Previous research had revealed that mutations in both *REV* and *KAN1* displayed reduced hypocotyl elongation in response to shade [18, 20, 30]. We therefore tested the hypocotyl elongation response of *wox4* mutant plants grown in white light (WL) and far-red enriched WL conditions (WL+FR) to simulate shade and trigger the shade avoidance response. We found that *wox4* mutants are indeed impaired in responding to shade and showed an even greater impairment of hypocotyl elongation than *rev-5* mutant plants (Fig 2A). As Arabidopsis plants require auxin production to respond to shade, loss-of-function mutations in the gene encoding the auxin biosynthesis enzyme TAA1 are almost completely insensitive to shade [8, 31, 32]. Using our experimental set-up, *taa1* mutant hypocotyls did not elongate when grown in WL+FR compared to WL conditions (Fig 2A). Similarly, to *wox4* and *rev-5* mutants, *taa1* mutants were indistinguishable from wild type plants, with respect to the length of the hypocotyl, when grown in WL conditions (Fig 2A). We also tested the response of *wox4 rev-5* double mutants in WL and WL+FR and found that respective double mutants resembled the *wox4* single mutant, indicating a redundant control of shade-induced growth (Fig 2A). Higher order mutations in *KANADI* genes, as in the *kan1 kan2* double mutant, also affected elongation growth, and these mutants displayed longer hypocotyls in WL conditions and shorter hypocotyls in WL+FR compared to the wild type (Fig 2A). In combination with *wox4*, *wox4 kan1 kan2* triple mutants had longer hypocotyls in WL when compared to the wild type, but elongated to a far lesser extent, when considering the absolute extension (subtraction of the length of the hypocotyl in WL from the length observed in WL+FR, Fig 2B). We confirmed that the changes in hypocotyl length we had observed were statistically significant, by performing two-way ANOVA analysis with all data (S3 Fig). The fact that *WOX4*, *KAN1* and *KAN2* encode transcriptional repressors, suggests that the expression of many target genes will be unleashed in *wox4 kan1 kan2* triple mutant plants and some of these de-repressed targets likely promote hypocotyl elongation in a synergistic fashion. Taken together, our findings indicated that REV and KAN1, KAN2 together with WOX4 are all required for an effective response to shade.

HD-ZIPIII activity is controlled at the post-translational level by the LITTLE ZIPPER3 (ZPR3) microProtein and plants overexpressing *ZPR3* are almost insensitive to shade with respect to their hypocotyl elongation ability [18]. We wondered if the role of WOX4 in the shade response could be related to the activity of the shoot apical meristem. Mutations in the gene encoding the WUSCHEL (WUS) transcription factor (here *wus-1*) showed a shoot meristem-less phenotype but when grown in shade conditions were able to induce hypocotyl growth comparable to wild type plants (S4 Fig). In comparison, transgenic *35S*::*ZPR3* plants showed a high frequency of shoot meristem arrests and the fraction of shoot meristem-less transgenic plants were strongly impaired in their ability to elongate the hypocotyl (S4 Fig). These findings indicated that a functional active shoot meristem was not required for the shade dependent hypocotyl elongation response, suggesting that HD-ZIPIII-mediated cell-fate changes within the hypocotyl might enable growth.

### Role of the REV/KAN1/*WOX4* module in shade-mediated tissue patterning

**Fig 3:**
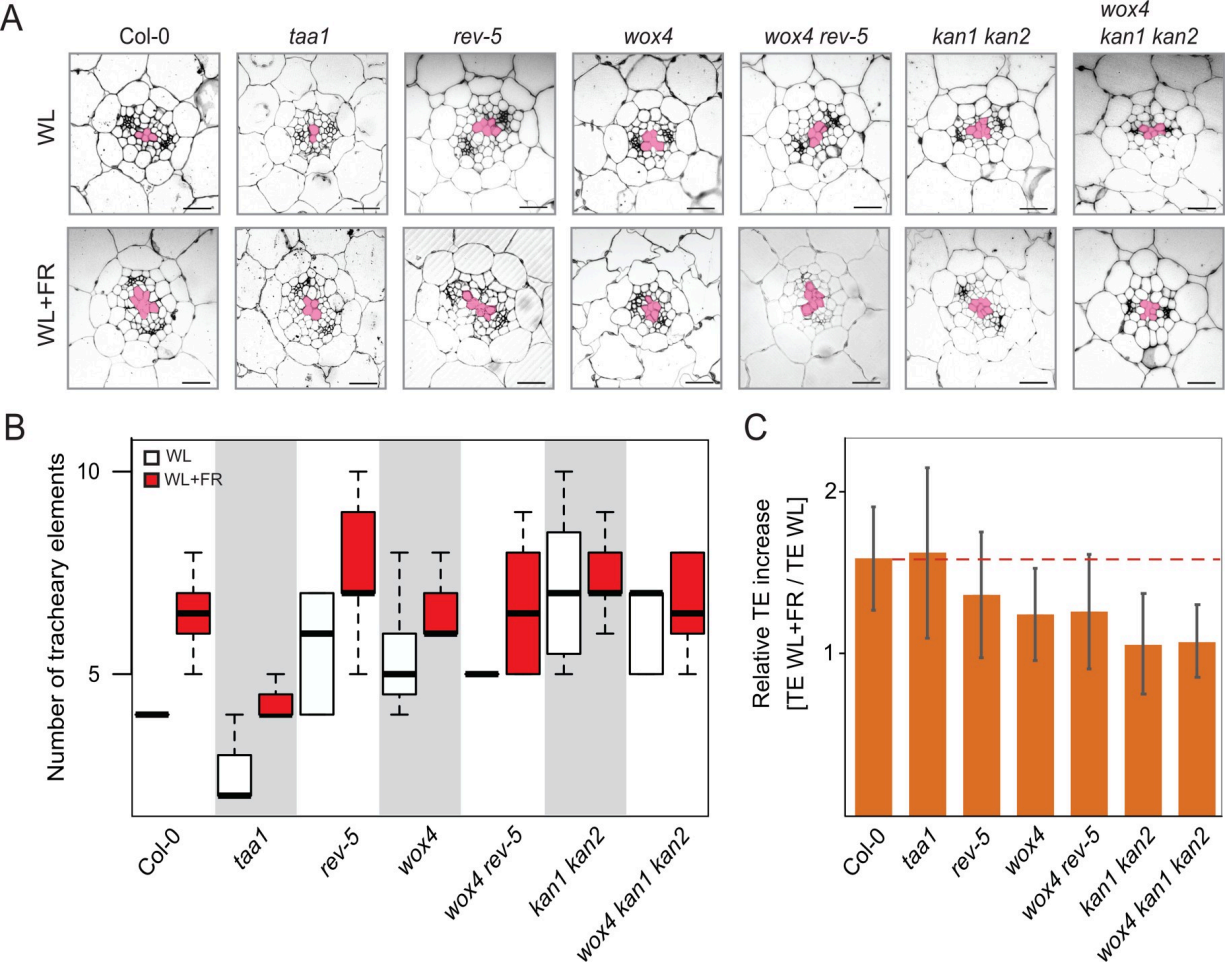
Shade-induced vascular patterning in mutants alters tracheary element formation. **A**, Representative images of hypocotyl cross sections of 10-day old seedlings grown in white light (WL) or shade (WL+FR) conditions. Pink colored areas mark the TE cells in the center of the vascular cylinder. Scale bars, 20 μm. **B**, Box plots show the observed experimental data of TE numbers in WL and W+FR conditions for wild type and different mutants. Shown is the average of two biological replicates n = 9–13. Except *taa1* which is based on one biological experiment n = 4–7. **C,** Relative TE increase by dividing the number of TEs in WL+FR with the number of TEs in WL. Red line indicates the ratio of the Col-0 wild type.

Given the role of *WOX4* in (pro)cambial cell proliferation [27, 28], it is conceivable that the dampened growth response of *wox4* mutant seedlings is caused by changes in vascular patterning. REV and KAN1, KAN2 have previously been implicated in vascular patterning as well [33–36] and we thus asked whether mutations in these genes would affect vascular responses in shade. We performed histological analyses to test whether we could observe differences in the vasculature of hypocotyls grown in WL and shade (WL+FR) conditions. When exposed to shade, Col-0 wild type plants displayed a highly reproducible increase in the number of tracheary elements in the vascular cylinder (Fig 3A and 3B). This increase also occurred in *taa1* mutant plants although the number of tracheary elements was strongly reduced compared to Col-0 wild type plants. Mutant plants *wox4*, *rev-5* and *wox4 rev-5* all exhibited a higher number of tracheary elements in WL conditions with only a moderate increase in WL+FR compared to Col-0 wild type plants (Fig 3A and 3B). We also examined *kan1 kan2* double and *wox4 kan1 kan2* triple mutants which all had more tracheary elements than the wild type in WL but showed no increase in WL+FR (Fig 3A–3C). The supernumerary tracheary elements appearing in response to shade in Col-0 wild type plants presented larger diameters/lumens (Fig 3A), suggesting that metaxylem-type cells were formed. These findings suggested that the increased production of tracheary elements was associated with hypocotyl elongation to confer a maximal shade avoidance response. To verify that the changes in tracheary element numbers we had observed were statistically significant, we performed two-way ANOVA analysis with all data shown in Fig 3 (S5 Fig). This analysis revealed that the observed changes in Col-0, *rev5*, *wox4* and *wox4 rev5* are significant (p-values between 0.05 and 0.001) and genotypes responded differently to treatments (ANOVA interaction term p-value <0.05). The comparisons of TE numbers in WL and WL+FR in *kan1 kan2* and *wox4 kan1 kan2* revealed no significant differences.

In Arabidopsis, phytochrome B is the major photoreceptor that in WL conditions represses hypocotyl elongation. Hence, mutations in *PHYB* result in a constitutive long hypocotyl phenotype [11]. We tested if the shade-insensitive *phyB-9* mutant plants, which do not elongate their hypocotyls in shade, would also be impaired in increasing tracheary element numbers. In agreement with our previous findings, we observed no supernumerary tracheary elements or changes in diameter/lumen in *phyB-9* mutant plants indicating that PHYB operates upstream of the REV/KAN1/WOX4 patterning module (S6 Fig).

To verify that the elongation defects we observed were not related to a general inability of vascular patterning mutants to elongate the hypocotyl, we investigated the role of brassinolide (BL) in promoting elongation growth. We grew wild type and mutant plants in white light conditions on plates containing either regular MS medium or MS with additional BL. As expected, all plants reacted to BL and showed hypocotyl elongation responses. The analysis of the vasculature revealed no increase in tracheary element numbers or diameter/lumen (S7 Fig). These findings revealed that the increase in tracheary element numbers and diameter/lumen is shade-induced and not required for elongation responses *per se*.

Several observations demonstrate that REV and WOX4 are required for a full shade avoidance response. First, we find that mutations in respective genes show a reduced hypocotyl elongation response (Fig 2A and 2B) and second, the mutants also fail to increase the number of TEs in response to shading (Fig 3A–3C). To test if shade-derived signals are not properly transported through the vascular system of *rev-5* and *wox4* mutant plants, we performed a 24-hour shade experiment. In this experiment, we exposed Col-0 wild type, *rev-5*, *wox4*, *kan1 kan2* and *taa1* mutants to WL+FR and determined hypocotyl growth after 24 hours. We found that all plants responded similar to WL+FR treatment and extended their hypocotyls except *taa1* mutant plants that only extended their hypocotyls weakly (S7 Fig). These findings indicate that *rev-5* and *wox4* mutant plants have no defect in detecting and responding to shade but fail to strongly elongate their hypocotyls in persistent shade.

**Fig 4:**
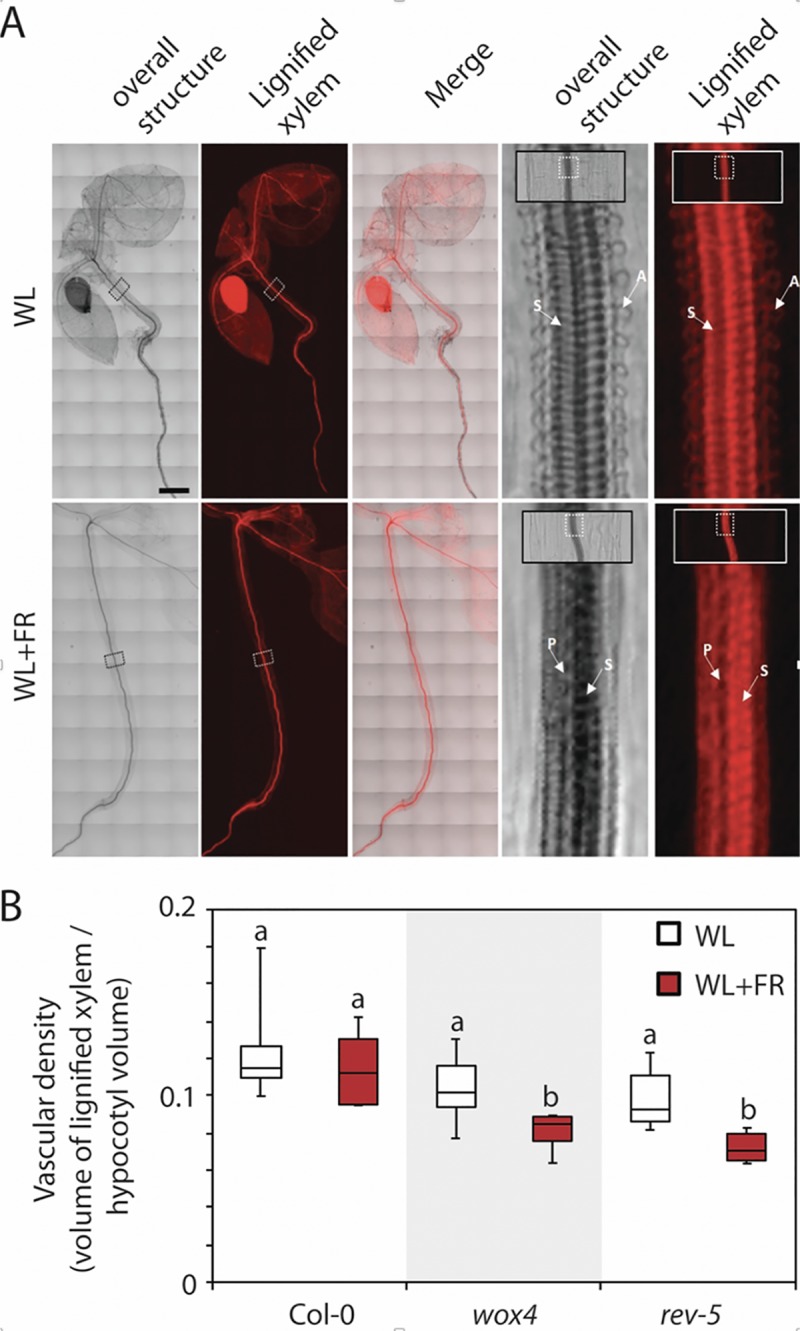
Virtual sections of white light and shade grown plants. **A**, Whole seedling tomography experiment with Col-0 wild type plants. Square boxes in the whole seedling images are enlarged to the right to highlight the organization of the vascular system. Arrows indicate tracheary elements with different secondary cell wall patterning: A for annular, S for spiral and P for pitted. Scale bar = 250 μm. **B**, Measurements of the vascular density defined by the volume of the lignified xylem divided by the hypocotyl volume. (n = 6 seedlings per genotype).

To further investigate the shade-induced vascular changes, we performed whole plant tomography experiments and virtually sectioned hypocotyls using confocal microscopy. In agreement with our histological analysis, we found that in wild type plants the increase of tracheary elements in the vascular cylinder of the hypocotyl in response to shade was associated with the appearance of pitted tracheary elements absent form WL (Fig 4A and S1 Movie). To evaluate how the supernumerary tracheary elements affected the overall hypocotyl structure in response to shade, tissue proportion analysis was performed using whole plant tomography in wild type, *wox4* and *rev5* to define if shade-dependent elongation altered the vascular density of plant tissue. Although significant increases could be detected for all genotypes tested between WL and WL+FR with regard to hypocotyl elongation, vascular density was unaltered in WL conditions but reduced in *wox4* and *rev5* specifically under WL+FR conditions (Fig 4B). This suggests that the increased tracheary element formation in response to shade is to maintain constant vascular density. The capacity of *wox4* and *rev5* to increase vascular density was reduced which was likely a result of lower vascular formation. In summary, our findings showed that long-term shading changed the composition of the vascular cylinder inside the hypocotyl resulting in an increased number of tracheary elements with larger diameter/lumen and different secondary cell wall organization to ensure that the vascular density of plant tissues are maintained. Mutations in either *REV*, *WOX4* or *KAN1* and *KAN2* genes caused a decrease in the plant capacity to increase the tracheary elements necessary to maintain the constant vascular density, thus demonstrating that these genes act as adaptive factors controlling the response to shade.

### Analysis of shade-induced vascular changes in crop plants

To further understand if the observed increase of tracheary elements in response to shade occurs only in Arabidopsis or can also be seen in other species, we decided to investigate different shade-sensitive food crops. For this, we tested tomato, carrot and dill seedlings, germinated them in WL and performed growth assays in WL and WL+FR conditions. In these experiments, we found that tomato, dill and carrot showed significant hypocotyl elongation responses, when grown in WL+FR conditions (S8 Fig). Histology of the hypocotyls revealed that tomato plants showed shade-related changes in vascular organization and shade treated plants produced around 20% more tracheary elements compared to the white light grown plants (S8 Fig). The supernumerary tracheary elements appearing in response to shade also presented larger diameters/lumens in tomato (S8 Fig). Similar changes were also observed in carrot seedlings that also showed a significant increase in tracheary elements in response to shading (S8 Fig). Dill seedlings showed hypocotyl elongation in response to shade but the number of tracheary elements was the same in WL and WL+FR (S8 Fig). These results indicated that the effect of increasing the number and size of tracheary elements in the hypocotyl of shade-responsive plants was not restricted to Arabidopsis and also occurred in economically important plants. The finding that not all species that responded to shade also showed extra tracheary elements indicated that species-specific plasticity responses exist.

### Analysis of differential gene expression in vascular patterning mutants in white light and shade

Based on the response to shade, we found that *REV* and *WOX4* genetically interact to promote growth in response to increased far-red light. To characterize respective mutants at the molecular level, we performed RNAseq to identify differentially expressed genes (DEGs). We found 140 DEGs in *rev-5* and 22 in *wox4* mutant plants. To assess which processes are mainly regulated by REV and WOX4, we performed gene ontology analyses with the individual sets of DEGs. Specifically, we found that DEGs in the *rev-5* mutant were related to secondary metabolism and responses to stress while the analysis of the 22 DEGs in *wox4* yielded no GO enrichment (S9 Fig). A comparison of the overlaps between the individual sets of DEGs revealed eleven genes that were upregulated in both *rev-5* and *wox4* mutants compared to wild type plants. Among these eleven upregulated transcripts we found the TRACHEARY ELEMENT DIFFERENTIATION RELATED7 (TED7) gene that plays a role in TE differentiation [37].

**Fig 5:**
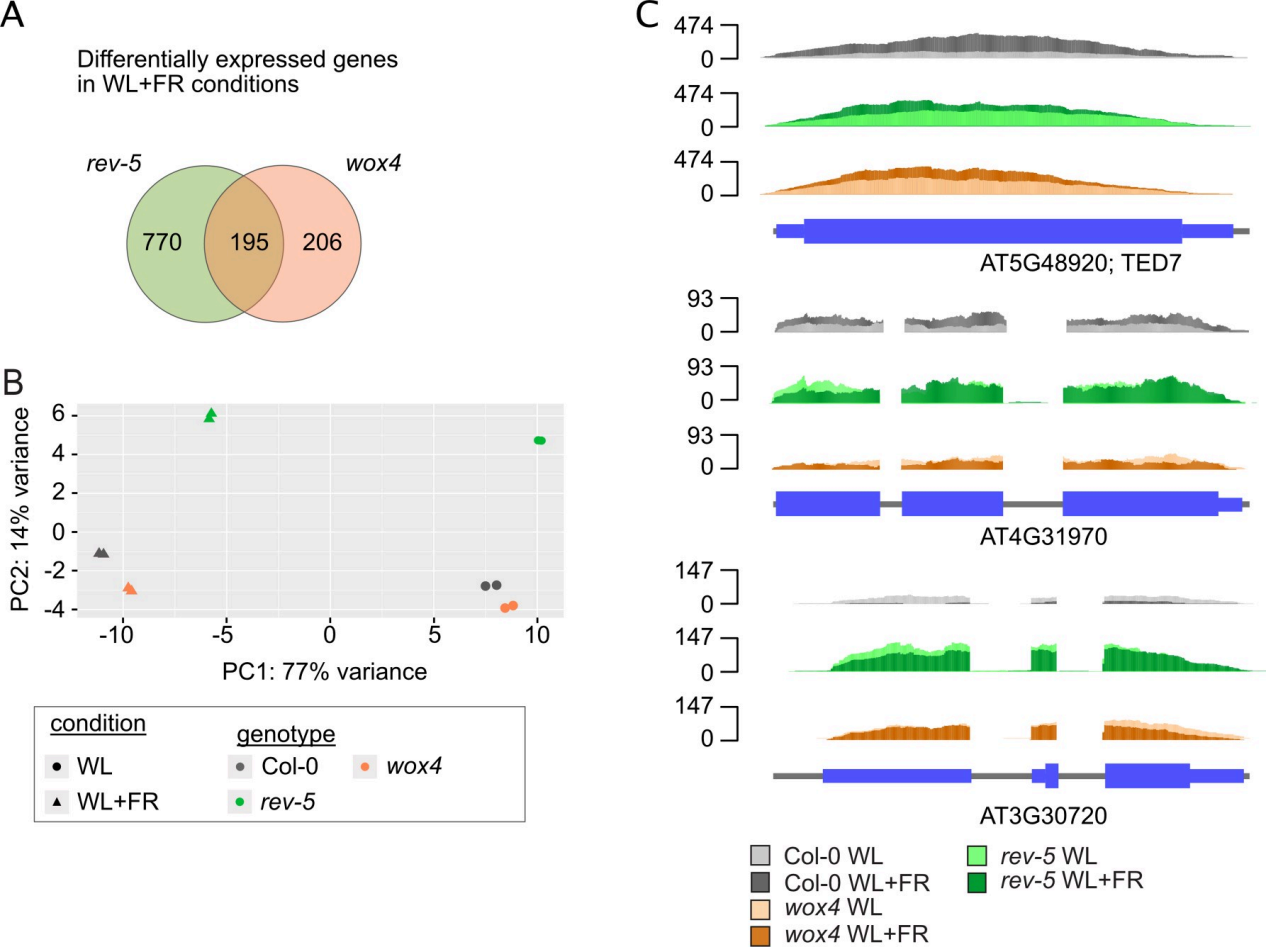
Shade-induced transcriptome changes in vascular patterning mutants. **A**, Venn diagram depicting differentially expressed genes (DEGs) in response to shade in the different genetic backgrounds (log2FC > +1/-1, BH-adj. p-value < 0.01). **B**, Principle component analysis of the gene expression (regularized logarithm transformed count data) between the different RNA-seq libraries. Plotted is the percentage of variance for each component. **C**, RNA-Seq read coverages of representative candidate genes showing the expression levels in the different genetic backgrounds. Upper panel shows a gene (*AT5G48920*; *TED7*) that is expressed at higher levels in WL in *rev-5* and *wox4*; the middle panel depicts a gene (*AT4G31970*) that is more robustly expressed in *rev-5* and much lower in *wox4* while the lower panel depicts a gene (*AT3G30720*) that is higher expressed in the mutant plants compared to wild type.

We next analyzed the changes of the transcriptomes of *rev-5* and *wox4* mutants when exposed to shade. As before, plants were cultivated for two days in white light conditions and then cultivated for an additional eight days in WL+FR. Transcriptome changes were compared to the control group of plants that was cultivated in WL for the entire ten days. To identify shade de-regulated genes globally, we compared the RNA-seq datasets using DESeq2 and used Col-0 as a reference (S1 Data). This analysis revealed in total 965 genes significantly deregulated transcripts in *rev-5* mutant plants and 401 in *wox4* mutants with an overlap of 195 genes (Fig 5A). Principle component analysis revealed that individual replicates grouped close together suggesting a high reproducibility of the data (Fig 5B). A more detailed analysis of de-regulated genes, especially the 195 genes that were found to be deregulated in all genotypes compared to wild type, revealed several genes encoding glucosinolate biosynthesis enzymes, cell wall modifying enzymes and genes involved in photosynthesis (S2 Data). This finding indicates that these mutants might also differ from wild type with regard to their metabolism and cell wall biochemistry. Investigation of specific transcripts that were differentially expressed in *wox4* and *rev-5* mutants showed that the *TED7* transcript is shade sensitive and expressed at higher levels in white light conditions in the two mutants. The gene encoding the QUA-QUINE STARCH enzyme was expressed at lower levels in *wox4* and higher levels in *rev-5* and in both genotypes the expression was no longer shade-inducible (Fig 5C). The CYP450 enzyme (*AT4G31970*) was ectopically high in both *wox4* and *rev-5* mutant plants (Fig 5C). Thus, the inability to induce genes in response to shade can result from elevated expression in white light conditions or a repression in shade.

### Far-red light has a transformative effect on in vitro inducible cell suspensions

Our work uncovered shade-induced changes mediated by a small set of patterning factors to promote the differentiation of cells into tracheary elements in whole seedlings. To elucidate whether far-red light itself possessed a transformative capacity, we investigated the effect of far-red irradiation on *in vitro* grown inducible hormone-habituated Arabidopsis cell suspension cultures. Addition of auxin, brassinosteroids and cytokinin in a specific ratio triggered the trans-differentiation of the actively dividing parenchyma into tracheary elements [38] which have the characteristic secondary cell wall patterns that can be recognized under a regular light microscope (S10 Fig) [39]. We investigated the influence of light quality on hormonal induction of xylogenesis and cell division. In response to white light, xylogenic induction was slightly reduced compared to induction in the dark or in white light supplemented with far-red light (S10 Fig) indicating that WL, or a high R:FR ratio suppressed xylogenesis. In contrast, WL also stimulated cell division compared to the dark and WL+FR (S10 Fig). In all conditions, the kinetics of tracheary element formation was not affected with cell differentiation starting at day 3 and plateauing by day 5. Interestingly, tracheary element cell wall organization changed with light conditions as WL+FR promoted reticulate-type tracheary elements in comparison to WL and dark. We were surprised to find that in the absence of the inducing hormone cocktail, far-red enriched light had a significant transformative capacity resulting in an increased frequency of spontaneous xylogenesis (S10 Fig). The latter effect was independent of increases in cell division (S10 Fig). These results complemented our previous findings by confirming that shade directly affected the vascular formation by promoting the differentiation of metaxylem tracheary elements. Moreover, our experiments using inducible cell cultures indicated that this shade-induced cell fate change occurred without undergoing cell division.

## Discussion

Compared to animals, plant development is highly plastic and the final shape of plants is strongly influenced by the environment. In the past decades, the genetic control required to respond to environmental inputs has been in part elucidated by the study of genes encoding master regulators of development as well as genes encoding transcription factors, photoreceptors or hormone biosynthetic enzymes. Knowledge on how existing patterning networks can be influenced by external signals have so far remained scarce.

Previous research identified several shade-related direct downstream targets genes of the patterning factors REVOLUTA and KANADI1 [18, 19, 24, 25, 40]. However, the role of REV and KAN1 in the control of growth in response to shade has been enigmatic. Our studies have revealed a role for *WOX4* in the control of growth in response to shade. Based on the phenotypes of loss-of-function mutants in *REV* (*rev-5*), *WOX4* (*wox4*) and the resultant double mutant (*wox4 rev-5*) we observed additive phenotypes with regard to the structures of the stem vasculature (S2 Fig) and an epistatic relationship in the control of growth in response to shade. In the latter process, *wox4* is epistatic over *rev-5*, as indicated by the *wox4*-like phenotype of the *wox4 rev-5* double mutant (Fig 2A and 2B). The *kan1 kan2* double mutant showed elongated hypocotyls in white light conditions and slightly longer hypocotyls in shade conditions but the relative growth in shade was reduced (Fig 2A and 2B). In combination with *wox4*, we found that *wox4 kan1 kan2* mutants showed even longer hypocotyls in both shade and non-shade conditions, supporting an additive relationship. The molecular function of WOX4 is still opaque and it is unclear what its direct downstream targets are. However, it is known that WOX4 affects the sensitivity of cells to auxin [28]. KAN1, KAN2 are known transcriptional repressors and WOX4 is predicted to act as a transcriptional repressor as well. Thus, it is possible that the combination of mutations (as in *wox4 kan1 kan2*) unleashes auxin production and signaling and thereby causes ectopic growth.

In a situation of enhanced KAN1 expression (Fig 1C), *WOX4* expression is abolished and transgenic plants overexpressing *KAN1* (*35S*::*KAN1*) are completely shade insensitive [40]. However, in comparison to *wox4* single mutants the growth phenotype of *35S*::*KAN1* is much stronger. The latter is likely attributed to KAN1 repressing a large number of target genes that are involved in hormone biology and shade-induced growth promotion [18, 19].

Importantly, with regards to their transcriptomes, *rev-5* mutants had more differentially expressed genes compared to *wox4* mutants (S9 Fig). Principle component analysis of the datasets revealed that *rev-5* mutant plants were markedly different from *wox4* and wild type plants but grouped with the different treatment categories (Fig 5B). Our analysis compared plants grown for eight days in WL+FR environment to plants grown in WL. This long-term exposure results in morphological changes such as petiole extension and leaf flattening. Some of the gene expression changes we observed might not relate to alterations in signaling but could be a consequence of above-mentioned morphological changes.

Our work revealed that Arabidopsis plants responded to shade by increasing the numbers and types of tracheary elements in the vascular cylinder of the hypocotyl to maintain a constant vascular density. Mutants that failed to increase tracheary element cell number showed reduced hypocotyl growth suggesting that plant growth adaptability depended on its capacity to maintain a constant vascular density. Analysis of genes differentially regulated in *rev-5* and *wox4* mutant plants revealed eleven genes that were upregulated in both mutant plants compared to Col-0 wild type plants (S9 Fig). This dataset comprised mostly uncharacterized enzymes and TED7.

**Fig 6:**
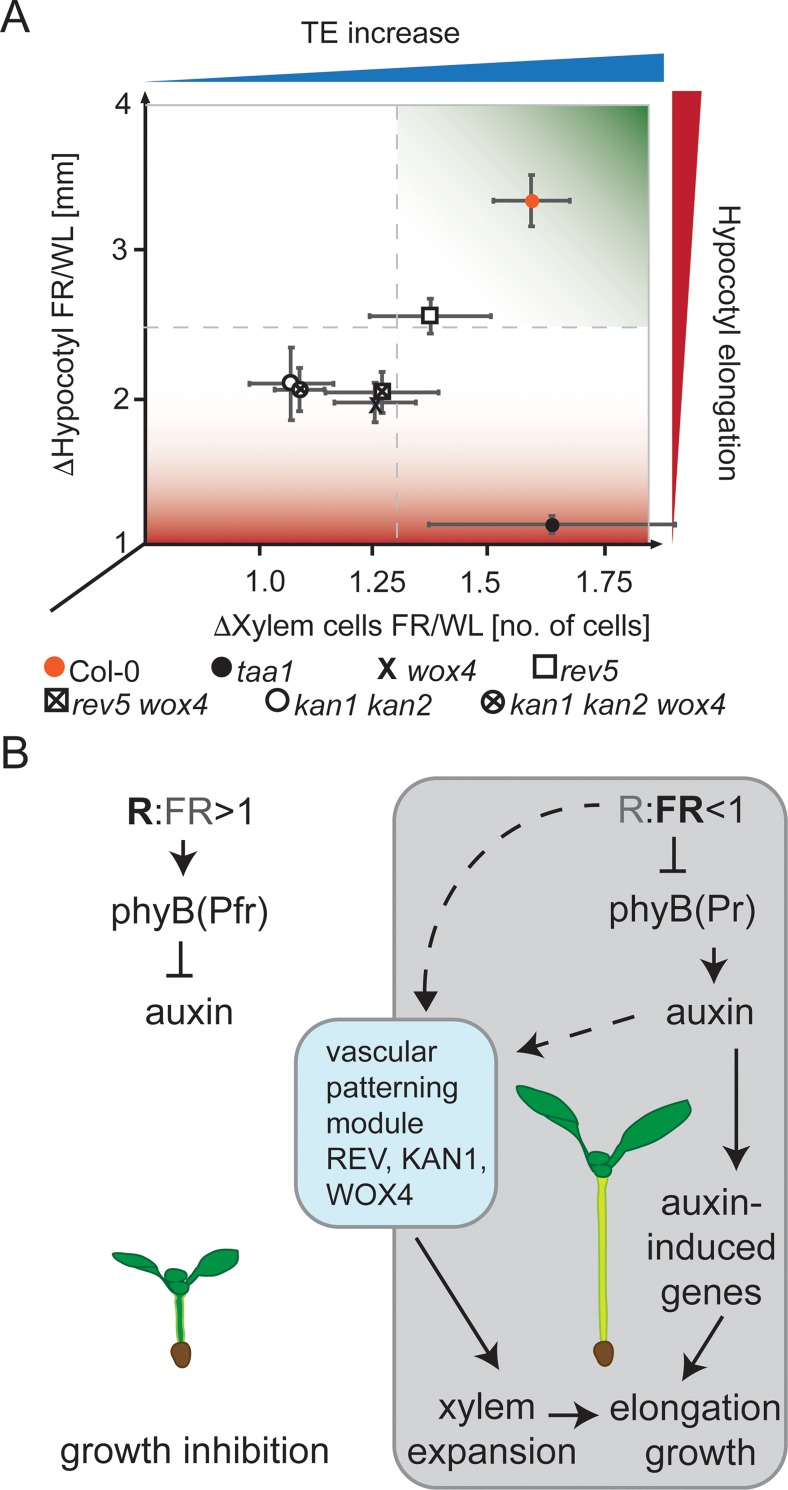
Differential growth analysis and model. **A**, Diagram plotting the relative extension of the hypocotyl (y-axis; length of the hypocotyl in WL+FR divided by the length in WL conditions) against the relative changes in TE cell number (x-axis; number of TE cells in WL+FR divided by the number of TE cells in WL conditions) including the added standard errors. **B**, Our model proposes that downstream of the perception of shade, a vascular patterning module operates to induce trans-differentiation of TE cells without new cell divisions. The increase in TE numbers is required to maintain constant vascular density in the hypocotyl during elongation. It is however possible that the auxin produced in response to phytochrome signaling impinges on the vascular patterning module and contributes to the increase in TE numbers.

To validate the findings that vascular patterning mutants showed less TE expansion in shade, we carried out a differential phenotype to genotype association analysis. Specifically, we determined if the observed changes in hypocotyl length and tracheary element increase were interdependent. We displayed the relative changes by dividing the length of the hypocotyl in WL with the length observed in WL+FR conditions plotted against the relative changes in tracheary elements. For the latter, we again divided the cell number in WL with the cell number in WL+FR. This analysis yielded direct associations between the relative length of the hypocotyl in relation to the relative increase of tracheary element numbers in shade conditions. Arabidopsis wild type plants which showed both a high plasticity in hypocotyl expansion and had increased numbers of tracheary elements, clustered as expected in the upper right corner of the diagram (Fig 6A). The *rev-5* mutant clustered in the middle of the diagram showing a weaker hypocotyl response and a slight increase in the numbers of tracheary elements in shade conditions. The *taa1* mutant clustered in the bottom right corner because it showed a relative increase in the number of tracheary elements in response to shading but failed to elongate the hypocotyl. All other mutants analyzed in this study clustered in the bottom left section of the diagram indicative of a dampened hypocotyl elongation response and concomitant failure to increase the number of tracheary elements in response to shade conditions. These results show that patterning factors of the HD-ZIPIII and KANADI families, including their direct downstream targets such as *WOX4* and *TAA1*, are required to promote shade-induced growth. To exclude a general defect of vascular patterning mutants to increase elongation growth we studied the effect of BL of inducing elongation growth and TE expansion (S7 Fig). We neither found an effect of BL in inducing TE expansion, nor were vascular patterning mutants impaired in their response to BL. Finally, the differential phenotype to genotype association analysis of the BL effect showed no major deviation of *taa1* and *wox4* mutant plants from the growth behavior of wild type plants (S11 Fig).

The shade avoidance response is a major determinant of how dense crops can be grown in a field. It is an undesirable trait because shade-sensitive plants redistribute resources from contributing to yield to sustaining excessive elongation growth [41]. Thus, a deeper understanding of the underlying transcription factor networks, hormonal networks and physiological responses will help to design future crops with uncoupled or dampened shade responses. We propose that in response to shade, a vascular patterning module composed of REV, KAN1 and WOX4 and maybe redundant transcription factors of these gene families, initiates differentiation inside the vascular cylinder leading to an increase in the number of tracheary elements (Fig 6B). The failure to strongly increase tracheary elements in shade could be related to reduced (pro)cambial proliferation of *wox4* mutant plants. We further showed that the increase of differentiation into tracheary elements occurs without the need for additional cell division, and that FR itself could promote cell trans-differentiation independently of auxin surplus (S10 Fig). As the observed increase in xylem maintains a constant vascular density, this might help in facilitating elongation growth by keeping surrounding tissues constantly hydrated. Furthermore, additional metaxylem-type tracheary elements with denser secondary cell walls might also add structural support for the extended hypocotyl.

In summary, our findings show that patterning factors direct changes in the organization of the vascular cylinder in response to shade. The contribution of these changes to the hypocotyl elongation response remains opaque because of the uncoupling of shade-regulated genes in the patterning mutants studied here. It is possible that some of the shade-regulated genes contribute to the vascular changes that we have identified but it is equally possible that parallel pathways are affected that contribute to elongation growth. Additional tissue-specific analysis of how the signal is perceived and transmitted will be required to elucidate further factors and their interconnections to fully understand the shade-induced differentiation response that we have uncovered.

## Materials & methods

### Plant material, treatments, and hypocotyl measurements

Mutant and transgenic plants that were used in this study have been described earlier: *kan1 kan2* [40]; *rev-5* (A260V) [42]; *wox4* (*wox4-1*, GABI_KAT_462GO1) [28]; *taa1* [8]; *wus-1* [43]; *MIR165a-OX* [44]; *ZPR3-OX* [45].

For histological analysis and hypocotyl measurements, wild-type and mutant plants were grown on Murashige and Skoog (MS) medium supplemented with vitamins, in a growth chamber (CLF Plant Climatics. model: SE-41LAR2) with continuous white light (Philips (master) TL-D 18W/840 5A) for 7 days (10 days for histological analysis), at 22°C. For shade avoidance conditions, 2-day old plants grown in white light were transferred to a far-red enriched compartment for 5 days (8 days for histological analysis). The WL+FR compartment was equipped with additional FR-LEDs (GroLED_N 20150409-TZI). Additional light parameters: PAR: 13 μmol/m^2^/s (in both WL and WL+FR). R/FR in WL: 7.7; in WL+FR: 0.2.

For hypocotyl measurements, seedlings were photographed and hypocotyls measured using IMAGEJ. Two-way ANOVA was performed using the software R (version 3.6.1, R Core Team, 2017).

For ChIP experiments and gene expression analysis, Col-0, and both *35S*::*FLAG-GR-REVd* and *35S*::*FLAG-GR-KAN1* transgenic plants were grown in liquid MS medium supplemented with vitamins for 15 days in continuous light and induced with 25 μM dexamethasone (DEX) for 60 min (*35S*::*FLAG-GR-REVd*) or 120 min (*35S*::*FLAG-GR-KAN1*) prior to harvesting.

### Histological analysis of hypocotyls

10-day old Arabidopsis seedlings were vacuum-infiltrated for 15 minutes, fixed for four hours in Karnovsky’s Fixative, and embedded in resin, according to Spurr’s procedure (Spurr, 1969). Hypocotyls were sectioned (2 μm) on a SuperNova Reichert-Jung microtome, stained with Toluidine Blue-O 0.05%, pH 4.4, and visualized in bright field using a Nikon Eclipse 80i Fluorescence microscope. TE elements were counted and two-way ANOVA was performed using the software R (version 3.6.1, R Core Team, 2017).

### Whole-seedling tomography

10-day old Arabidopsis seedlings were fixed in 70% ethanol overnight and cleared using 10% NaOH solution at 60°C for 14 h. Cleared seedling were then staining with an 0.01% basic Fuchsin solution (857343, Sigma-Aldrich) in water for 1 h at 60°C, washed twice in water and mounted between glass and coverslip in 50% glycerol. Whole seedling tomography was then acquired using LSM510 meta confocal microscope (Zeiss, Sweden) equipped with an automated xyz stage, long working distance 20x objective and image stitching option. Whole seedling tomography resulted from stitching 50–60 xyz confocal stacks of 20–25 xy images (1024x1024 pixels) separated by 0.9 μm z averaged 3 times. Images were acquired using a 488 nm Ag laser excitation and collection emitted photon using a long pass 500 nm filter. Hypocotyl total and vascular volumes were measured by summing the respective areas in each optical section using IMAGEJ.

### Inducible cell suspension cultures

Arabidopsis thaliana Col-0 hormone-habituated cell suspension cultures were generated as previously described by Pesquet et al. [46]. Cell suspensions, submitted or not to xylogenic differentiation using a combination of auxin/cytokinin/brassinosteroids [38], were grown under 120 rpm orbital agitation at 25–27°C either in dark or 30 μE white light supplemented or not with far-red. After 7-days of culture, cell growth and tracheary element differentiation were measured as previously reported by Ménard et al., (2017). Similarly, tracheary element differentiation time-course and secondary cell wall organization was measured as previously reported by Derbyshire et al., (2015).

### Gene expression analysis

For gene expression analysis RNA was extracted using EURx GeneMATRIX Universal RNA Purification Kit. Purified RNA (1 μg) was used for reverse transcription using ThermoScientific Revert Aid Reverse Transcriptase with oligo-dT primers. Real-time quantitative PCRs (RT-qPCR) were carried out using the ThermoScientific SYBR Green qPCR master mix on a Biorad CFX384. Gene expression levels were calculated using the delta-Ct method and a standard curve relative to GAPDH. Oligonucleotide sequences are listed in S1 Table.

### RNAseq analysis

Respective seedlings were grown on MS plates for two days in white light conditions and then a fraction was transferred to a white light compartment with additional far-red light for eight days. RNA from two biological replicates were extracted for each genotype and treatment as described earlier and Illumina sequencing libraries were constructed (TruSeq) and sequenced on an Illumina HiSeq2000 platform (Novogene, Hongkong). Between 21 and 24 million read pairs per sample were obtained. Paired-end reads were loaded into Galaxy version 15.05.rc1 [47–49], and quality was assessed using FastQC (version 0.10.1). HISAT2 aligned above 85% of read pairs of each sample correctly to the Arabidopsis genome (TAIR10). To identify shade regulated transcriptome changes we used DESeq2 v1.18.1 in R v3.4.4, the interaction term for the light effect in mutant genotypes vs genotype Col-0 were calculated using the design "~ genotype + light + genotype:light" with Col-0 as reference. This tests if the light effect is different in mutant compared to Col-0. Raw data files are available through Gene Expression Omnibus (GSE137009).

### ChIP-qPCR analysis

ChIP experiments were carried out as described by Kwon et al. (2005) using Col-0, and transgenic 35S::FLAG-GR-REVd and 35S::FLAG-GR-KAN1 plants. Anti-FLAG M2 magnetic beads (Sigma) were used. After Cross-linking, DNA was purified with Qiagen MinElute PCR Purification kit. DNA was used for subsequent RT-qPCR.

## Supporting information

S1 Fig Shade leads to a reorganization of the vasculature in petioles. **A**, Organization of the vasculature in white light (left panel) and in shade (right panel). Upper panel: microscopic image of cross section of petioles, lower panel: cartoon of the boxed area in the cross section. Grey cells: ground tissue; blue cells: xylem; red cells: cambium; green cells: phloem. **B**, Analysis of the cambial marker *WOX4* (*pWOX4*::*GUS*) in white light and shade conditions.
(PDF)

S2 Fig Histological analysis of the stem base of *rev* and *wox4* mutant plants. **A**, Schematic overview of the arrangement of vascular tissue in the Col-0 wild type plant. **B**, Col-0 wild type, **C**, *35S*::*WOX4*, **D**, *wox4*, **E**, *rev-5*, **F**, *wox4 rev-5* double mutant plants grown in log day conditions. Main stems of plants were sectioned after reaching a minimal height of around 10 cm. Highlighted in red is the cambial cell layer.
(PDF)

S3 Fig Statistical analysis of shade-induced hypocotyl responses. **A**, Matrix depicting statistical significance scores using students T-test. Colors represent respective p-values: Red: p<0.001; orange: p<0.01; yellow: p<0.05; grey: not significant; white: not tested. **B**, Two-way ANOVA was carried out to test significance of genotypes, treatments and genotype:treatment interaction. Asterisks plotted by R. The interaction is significant and genotypes responded differently to treatments (ANOVA interaction term p-value <0.05).
(PDF)

S4 Fig Shade avoidance responses of mutants lacking a shoot apical meristem. **A**, Picture of representative seedlings overexpressing the ZPR3 microProtein (*35S*::*ZPR3*) and showing a meristem arrest phenotype in comparison to Col-0 grown in white light and shade (left panel); and meristem-less *wus-1* mutants and the corresponding L*er* wild type grown in white light and shade (right panel). **B**, Quantification of the hypocotyl length. Plotted is the average +/- SD. **C**, Ratio of the hypocotyl length in shade divided by the length in white light shows the reduced shade response of *35S*::*ZPR3* seedlings compared to Col-0 and the normal response of *wus-1* compared to L*er*.
(PDF)

S5 Fig Statistical analysis of shade-induced tracheary element (TE) numbers. **A**, Matrix depicting statistical significance scores using students T-test. Colors represent respective p-values: Red: p<0.001; orange: p<0.01; yellow: p<0.05; grey: not significant; white: not tested. **B**, Two-way ANOVA was carried out to test significance of genotypes, treatments and genotype:treatment interaction. Asterisks plotted by R. The interaction is significant and genotypes responded differently to treatments (ANOVA interaction term p-value <0.05).
(PDF)

S6 Fig Histological analysis of shade-induced vascular patterning in wild type and *phyB-9* mutant plants. **A**, Representative images of hypocotyl cross sections of 10-day old seedlings grown in both white light (WL) and shade (WL+FR) conditions. Pink colored areas mark the TE cells in the center of the vascular cylinder. **B**, Quantification of tracheary elements of one biological replicate. Plotted are averages +/- standard deviation, n = 5–6.
(PDF)

S7 Fig Histological analysis of brassinolide-induced vascular patterning. **A**, Representative images of hypocotyl cross sections of 10- day old seedlings grown in white light (WL) +/- brassinolide (BL). Pink colored areas mark the TE cells in the center of the vascular cylinder. Scale bars, 20 μm. **B**, Box plots show the observed experimental data of TE numbers in WL +/- BL for wild type, *taa1* and *wox4* mutant plants. Shown is one of three biological replicates n = 5–8. **C**, Box plots depicting the hypocotyl length; white boxes hypocotyls grown in WL-BL; red boxes, hypocotyls grown in WL+BL. **D**, 24-hour shade experiment. Plotted are hypocotyl lengths in WL and WL+FR conditions (shaded areas). *P<0.05; ***P<0.0005, determined by student’s t-test.
(PDF)

S8 Fig Hypocotyl response to shade in selected crop plants. **A**, Picture of representative seedlings grown in WL and WL+FR conditions. **B**, Quantification of the hypocotyl length of seedlings grown in WL and WL+FR conditions. **C-E**, Representative images of hypocotyl cross sections of 10-day old **C**, tomato, **D**, carrot and **E**, dill seedlings grown in white light (WL) and shade (W+FR) conditions. Box plots show the observed experimental data of TE numbers. T-Tests *p≤ 0.05, ** p≤ 0.005, ***p≤0.001.
(PDF)

S9 Fig Identification of differentially expressed genes in *rev5*, *wox4* and *wox4 rev5* mutant plants. **A**, top: Volcano plot showing differentially expressed genes in *rev5* compared to the Col-0 wild type. Bottom: gene ontology analysis using the agrigo tool showing that genes related to secondary metabolism and stress responses are over-represented. **B**, Volcano plot of *wox4* mutants compared to wild type; no GO enrichment was observed. **C,** Venn diagram showing the overlap of the transcriptome analysis (log2FC > +1/-1; BH-adj p-value <0.001).
(PDF)

S10 Fig Trans-differentiation of TEs *in vitro*. **A**, images of habituated cell culture cells in the non-differentiated state and TE differentiated cells after hormonal induction. Scale bars = 12μm. **B**, analysis of TE differentiation efficiency in different light qualities. **C**, determination of the cell density in induced conditions for different light regimes. **D**, Analysis of spontaneous TE differentiation in different light conditions. **E**, determination of the cell density of cells grown in different light regimes in the absence of inducing hormones. d = dark, w = white light, fr = white light supplemented with far-red light (n = 4 replicates of 1–2 experiments).
(PDF)

S11 Fig Differential growth analysis of plants grown on medium +/- brassinolide. Diagram plotting the relative extension of the hypocotyl (y-axis; length of the hypocotyl in WL+BL divided by the length in WL-BL conditions) against the relative changes in TE cell number (x-axis; number of TE cells in WL+BL divided by the number of TE cells in WL-BL conditions) including the added standard errors.
(PDF)

S1 Data Differentially expressed genes in shade identified using DESEq2 (WL versus WL+FR in *rev5* and *wox4* mutant plants using the design "~ genotype + light + genotype:light" with Col-0 as reference). (XLSX)

S2 Data Individual and overlapping datasets of differentially expressed genes in shade. (XLSX)

S1 Table Oligonucleotide sequences. (XLSX)

S1 Movie Sample movie of Col-0 (brightfield transmission) using xyz confocal optical sectioning and 3D-reconstruction with 180-degree rotation. Scale bar 500 μM.
(AVI)
