# Supplementary material for: Light affects tissue patterning of the hypocotyl in the shade-avoidance response
Source: PLoS Genet. 2020 Mar 23;16(3):e1008678. doi: 10.1371/journal.pgen.1008678 (PMC7153905; doi:10.1371/journal.pgen.1008678)
Supplement: S1 Fig — A, Organization of the vasculature in white light (left panel) and in shade (right panel). Upper panel: microscopic image of cross section of petioles, lower panel: cartoon of the boxed area in the cross section. Grey cells: ground tissue; blue cells: xylem; red cells: cambium; green cells: phloem. B, Analysis of the cambial marker WOX4 (pWOX4::GUS) in white light and shade conditions. (PDF) [file pgen.1008678.s001.pdf]

Botterweg et al.

Supplementary Figure S1

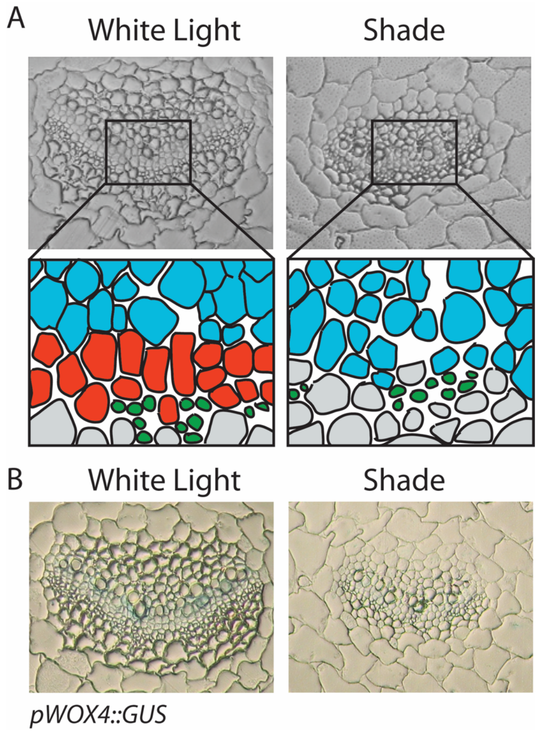


**Figure S1. Shade leads to a reorganization of the vasculature in petioles. A**, Organization of the vasculature in white light (left panel) and in shade (right panel). Upper panel: microscopic image of cross section of petioles, lower panel: cartoon of the boxed area in the cross section. Grey cells: ground tissue; blue cells: xylem; red cells: cambium; green cells: phloem. **B**, Analysis of the cambial marker WOX4 (pWOX4::GUS) in white light and shade conditions.
