# Supplementary material for: Light affects tissue patterning of the hypocotyl in the shade-avoidance response
Source: PLoS Genet. 2020 Mar 23;16(3):e1008678. doi: 10.1371/journal.pgen.1008678 (PMC7153905; doi:10.1371/journal.pgen.1008678)
Supplement: S2 Fig — A, Schematic overview of the arrangement of vascular tissue in the Col-0 wild type plant. B, Col-0 wild type, C, 35S::WOX4, D, wox4, E, rev-5, F, wox4 rev-5 double mutant plants grown in log day conditions. Main stems of plants were sectioned after reaching a minimal height of around 10 cm. Highlighted in red is the cambial cell layer. (PDF) [file pgen.1008678.s002.pdf]

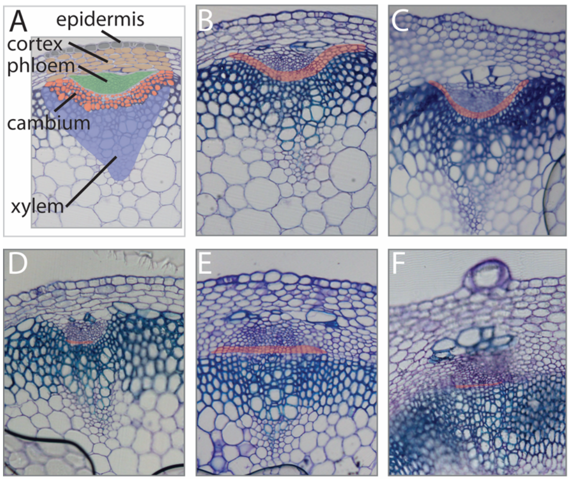

Figure S2. Histological analysis of the stem base of *rev5* and *wox4* mutant plants. A, Schematic overview of the arrangement of vascular tissue in the Col-0 wild type plant. **B**, Col-0 wild type, **C**, *35S::WOX4*, **D**, *wox4*, **E**, *rev-5*, **F**, *wox4 rev-5* double mutant plants grown in log day conditions. Main stems of plants were sectioned after reaching a minimal height of around 10 cm. Highlighted in red is the cambial cell layer.
