# Supplementary material for: Light affects tissue patterning of the hypocotyl in the shade-avoidance response
Source: PLoS Genet. 2020 Mar 23;16(3):e1008678. doi: 10.1371/journal.pgen.1008678 (PMC7153905; doi:10.1371/journal.pgen.1008678)
Supplement: S3 Fig — A, Matrix depicting statistical significance scores using students T-test. Colors represent respective p-values: Red: p<0.001; orange: p<0.01; yellow: p<0.05; grey: not significant; white: not tested. B, Two-way ANOVA was carried out to test significance of genotypes, treatments and genotype:treatment interaction. Asterisks plotted by R. The interaction is significant and genotypes responded differently to treatments (ANOVA interaction term p-value <0.05). (PDF) [file pgen.1008678.s003.pdf]

Botterweg et al.

Supplementary Figure S3

A

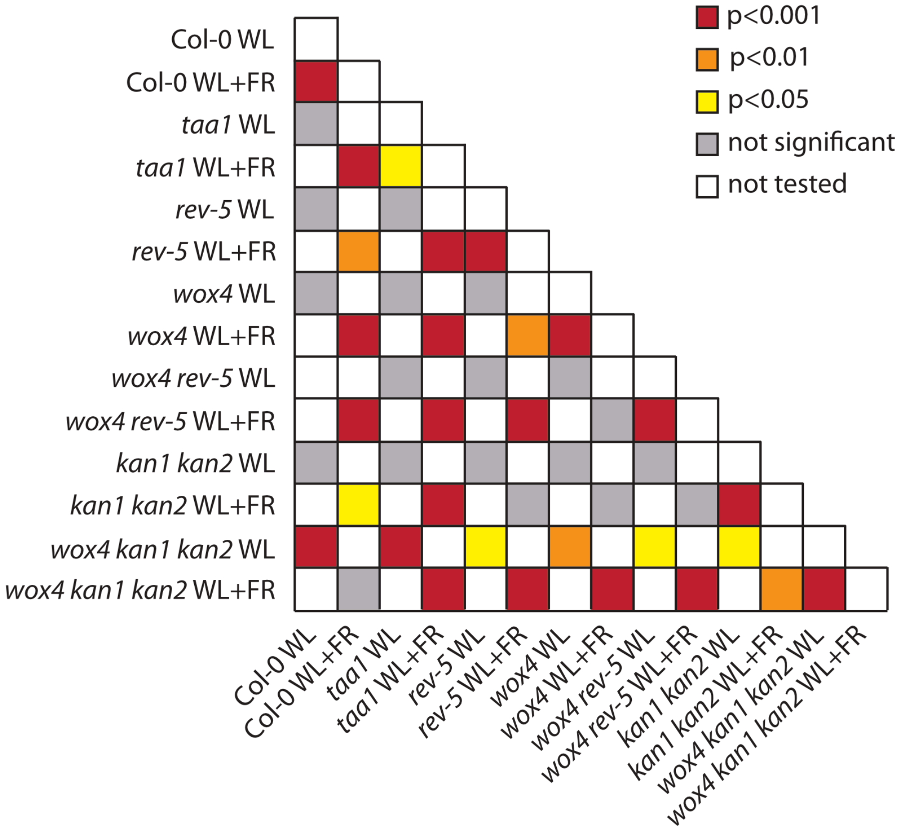

B

	Df	Sum Sq	Mean Sq	F value	Pr(>F)	
geno	6	22777	3796	23.109	<2e-16	***
treat	1	60500	60500	368.289	<2e-16	***
geno:treat	6	8072	1345	8.189	3.68e07	***
residuals	96	15770	164			

Supplementary Figure S3. Statistical analysis of shade-induced hypocotyl responses. A, Matrix depicting statistical significance scores using students T-test. Colors represent respective p-values: Red: p<0.001; orange: p<0.01; yellow: p<0.05; grey: not significant; white: not tested. **B**, Two-way ANOVA was carried out to test significance of genotypes, treatments and genotype:treatment interaction. Asterisks plotted by R. The interaction is significant and genotypes responded differently to treatments (ANOVA interaction term p-value <0.05).
