# Supplementary material for: Light affects tissue patterning of the hypocotyl in the shade-avoidance response
Source: PLoS Genet. 2020 Mar 23;16(3):e1008678. doi: 10.1371/journal.pgen.1008678 (PMC7153905; doi:10.1371/journal.pgen.1008678)
Supplement: S4 Fig — A, Picture of representative seedlings overexpressing the ZPR3 microProtein (35S::ZPR3) and showing a meristem arrest phenotype in comparison to Col-0 grown in white light and shade (left panel); and meristem-less wus-1 mutants and the corresponding Ler wild type grown in white light and shade (right panel). B, Quantification of the hypocotyl length. Plotted is the average +/- SD. C, Ratio of the hypocotyl length in shade divided by the length in white light shows the reduced shade response of 35S::ZPR3 seedlings compared to Col-0 and the normal response of wus-1 compared to Ler. (PDF) [file pgen.1008678.s004.pdf]

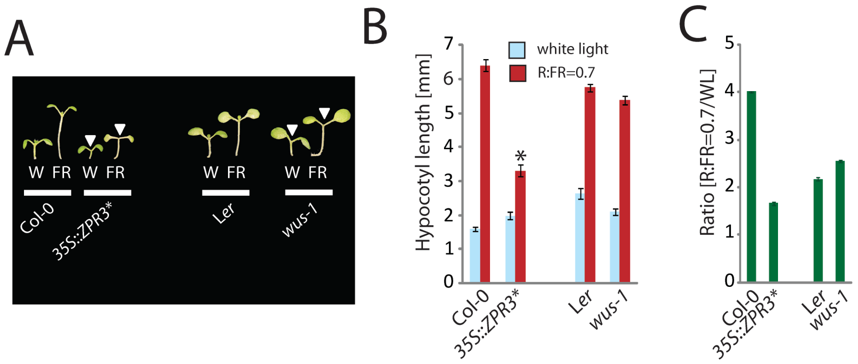

Figure S4. Shade avoidance responses of mutants lacking a shoot apical meristem. A, Picture of representative seedlings overexpressing the ZPR3 microProtein (*35S::ZPR3*) and showing a meristem arrest phenotype in comparison to Col-0 grown in white light and shade (left panel); and meristem-less *wus-1* mutants and the corresponding Ler wild type grown in white light and shade (right panel). **B**, Quantification of the hypocotyl length. Plotted is the average +/- SD. C, Ratio of the hypocotyl length in shade divided by the length in white light shows the reduced shade response of *35S::ZPR3* seedlings compared to Col-0 and the normal response of *wus-1* compared to Ler.
