# Supplementary material for: Light affects tissue patterning of the hypocotyl in the shade-avoidance response
Source: PLoS Genet. 2020 Mar 23;16(3):e1008678. doi: 10.1371/journal.pgen.1008678 (PMC7153905; doi:10.1371/journal.pgen.1008678)
Supplement: S5 Fig — A, Matrix depicting statistical significance scores using students T-test. Colors represent respective p-values: Red: p<0.001; orange: p<0.01; yellow: p<0.05; grey: not significant; white: not tested. B, Two-way ANOVA was carried out to test significance of genotypes, treatments and genotype:treatment interaction. Asterisks plotted by R. The interaction is significant and genotypes responded differently to treatments (ANOVA interaction term p-value <0.05). (PDF) [file pgen.1008678.s005.pdf]

A

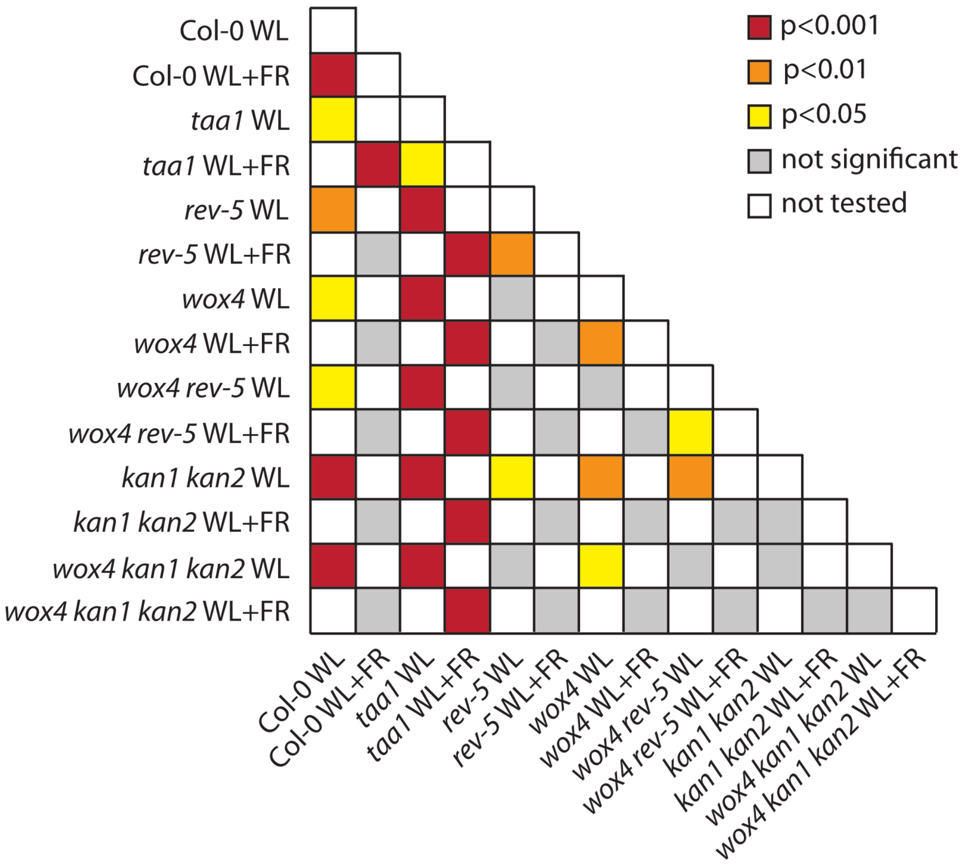

B

	Df	Sum sq	Mean Sq	F value	Pr(>F)	
geno	6	128.61	21.43	16.102	7.67e-14	***
treat	1	74.47	74.47	55.945	9.30e-12	***
geno:treat	6	21.21	3.53	2.656	0.0183	*
residuals	132	175.71	1.33			

Figure S5. Statistical analysis of shade-induced tracheary element (TE) numbers. A, Matrix depicting statistical significance scores using students T-test. Colors represent respective p-values: Red: p<0.001; orange: p<0.01; yellow: p<0.05; grey: not significant; white: not tested. **B**, Two-way ANOVA was carried out to test significance of genotypes, treatments and genotype:treatment interaction. Asterisks plotted by R. The interaction is significant and genotypes responded differently to treatments (ANOVA interaction term p-value <0.05).
