# Supplementary material for: Light affects tissue patterning of the hypocotyl in the shade-avoidance response
Source: PLoS Genet. 2020 Mar 23;16(3):e1008678. doi: 10.1371/journal.pgen.1008678 (PMC7153905; doi:10.1371/journal.pgen.1008678)
Supplement: S6 Fig — A, Representative images of hypocotyl cross sections of 10-day old seedlings grown in both white light (WL) and shade (WL+FR) conditions. Pink colored areas mark the TE cells in the center of the vascular cylinder. B, Quantification of tracheary elements of one biological replicate. Plotted are averages +/- standard deviation, n = 5–6. (PDF) [file pgen.1008678.s006.pdf]

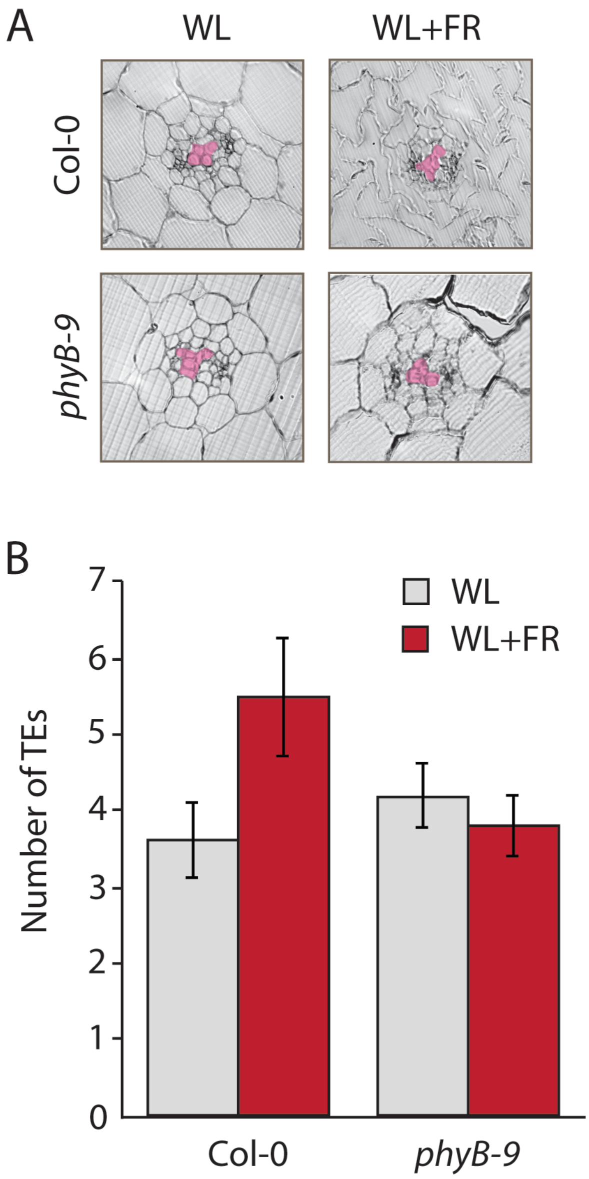

Supplementary Figure S6. Histological analysis of shade-induced vascular patterning in wild type and *phyB-9* mutant plants. A, Representative images of hypocotyl cross sections of 10-day old seedlings grown in both white light (WL) and shade (WL+FR) conditions. Pink colored areas mark the TE cells in the center of the vascular cylinder. **B**, Quantification of tracheary elements of one biological replicate. Plotted are averages +/- standard deviation, n=5-6.
