# Supplementary material for: Light affects tissue patterning of the hypocotyl in the shade-avoidance response
Source: PLoS Genet. 2020 Mar 23;16(3):e1008678. doi: 10.1371/journal.pgen.1008678 (PMC7153905; doi:10.1371/journal.pgen.1008678)
Supplement: S7 Fig — A, Representative images of hypocotyl cross sections of 10- day old seedlings grown in white light (WL) +/- brassinolide (BL). Pink colored areas mark the TE cells in the center of the vascular cylinder. Scale bars, 20 μm. B, Box plots show the observed experimental data of TE numbers in WL +/- BL for wild type, taa1 and wox4 mutant plants. Shown is one of three biological replicates n = 5–8. C, Box plots depicting the hypocotyl length; white boxes hypocotyls grown in WL-BL; red boxes, hypocotyls grown in WL+BL. D, 24-hour shade experiment. Plotted are hypocotyl lengths in WL and WL+FR conditions (shaded areas). *P<0.05; ***P<0.0005, determined by student’s t-test. (PDF) [file pgen.1008678.s007.pdf]

Supplementary Figure S7

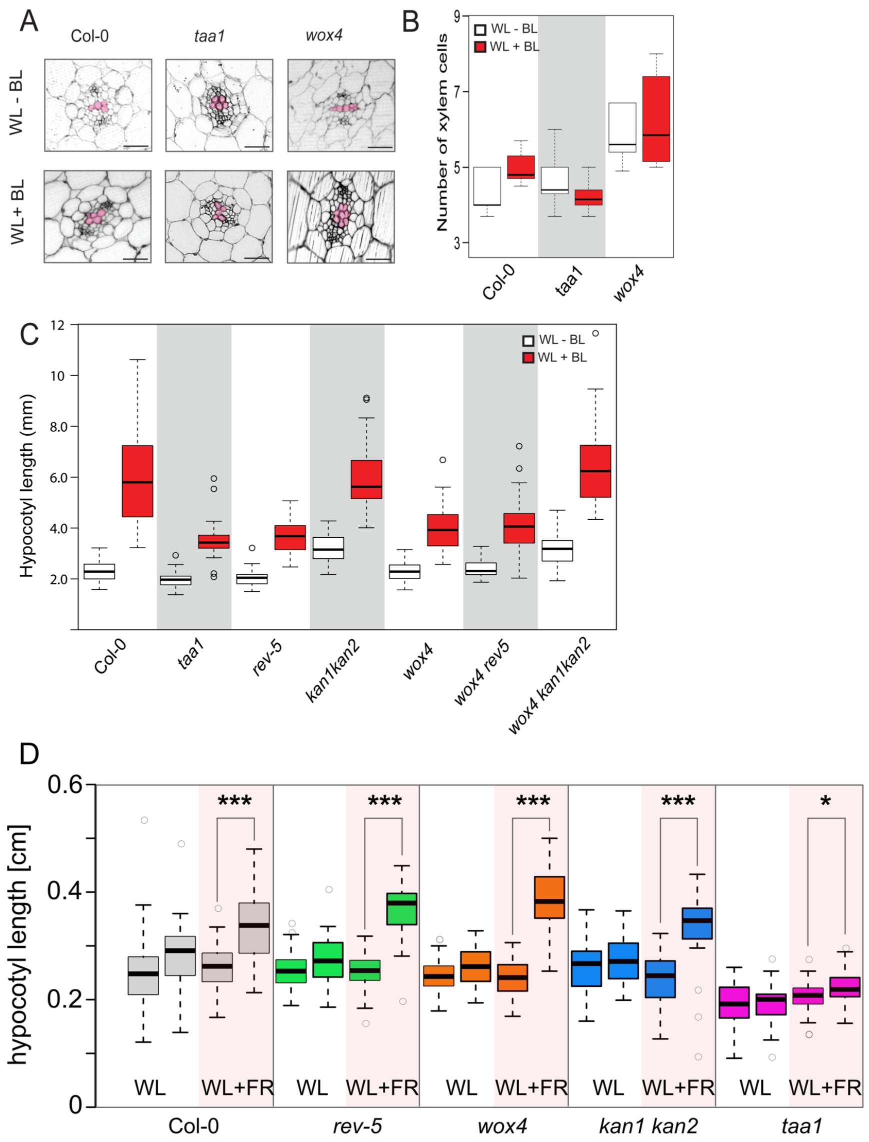

Supplementary Figure S7. Histological analysis of brassinolide-induced vascular patterning. A, Representative images of hypocotyl cross sections of 10-day old seedlings grown in white light (WL) +/- brassinolide (BL). Pink colored areas mark the TE cells in the center of the vascular cylinder. Scale bars, 20 µm. **B**, Box plots show the observed experimental data of TE numbers in WL +/- BL for wild type, *taa1* and *wox4* mutant plants. Shown is one of three biological replicates n=40. **C**, Box plots depicting the hypocotyl length; white boxes hypocotyls grown in WL-BL; red boxes, hypocotyls grown in WL+BL. **D,** 24-hour shade experiment. Plotted are hypocotyl lengths in WL and WL+FR conditions (shaded areas). *P<0.05; ***P<0.0005, determined by student's t-test.
