# Supplementary material for: Light affects tissue patterning of the hypocotyl in the shade-avoidance response
Source: PLoS Genet. 2020 Mar 23;16(3):e1008678. doi: 10.1371/journal.pgen.1008678 (PMC7153905; doi:10.1371/journal.pgen.1008678)
Supplement: S8 Fig — A, Picture of representative seedlings grown in WL and WL+FR conditions. B, Quantification of the hypocotyl length of seedlings grown in WL and WL+FR conditions. C-E, Representative images of hypocotyl cross sections of 10-day old C, tomato, D, carrot and E, dill seedlings grown in white light (WL) and shade (W+FR) conditions. Box plots show the observed experimental data of TE numbers. T-Tests *p≤ 0.05, ** p≤ 0.005, ***p≤0.001. (PDF) [file pgen.1008678.s008.pdf]

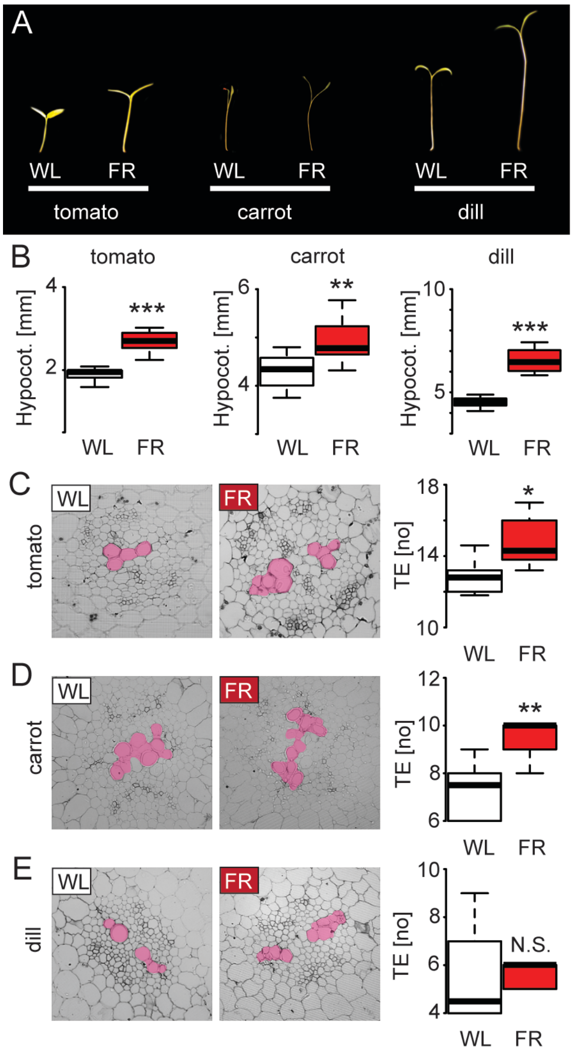


**Supplementary Figure S8. Hypocotyl response to shade in selected crop plants.**
**(A)** Picture of representative seedlings grown in WL and WL+FR conditions.
**(B)** Quantification of the hypocotyl length of seedlings grown in WL and WL+FR conditions.
**(C-E)** Representative images of hypocotyl cross sections of 10-day old C, tomato,
**(D)** carrot and
**(E)** dill seedlings grown in white light (WL) and shade (W+FR) conditions. Box plots show the observed experimental data of TE numbers. T-Tests *p≤ 0.05, ** p≤ 0.005, ***p≤0.001.
