# Supplementary material for: Light affects tissue patterning of the hypocotyl in the shade-avoidance response
Source: PLoS Genet. 2020 Mar 23;16(3):e1008678. doi: 10.1371/journal.pgen.1008678 (PMC7153905; doi:10.1371/journal.pgen.1008678)
Supplement: S9 Fig — A, top: Volcano plot showing differentially expressed genes in rev5 compared to the Col-0 wild type. Bottom: gene ontology analysis using the agrigo tool showing that genes related to secondary metabolism and stress responses are over-represented. B, Volcano plot of wox4 mutants compared to wild type; no GO enrichment was observed. C, Venn diagram showing the overlap of the transcriptome analysis (log2FC > +1/-1; BH-adj p-value <0.001). (PDF) [file pgen.1008678.s009.pdf]

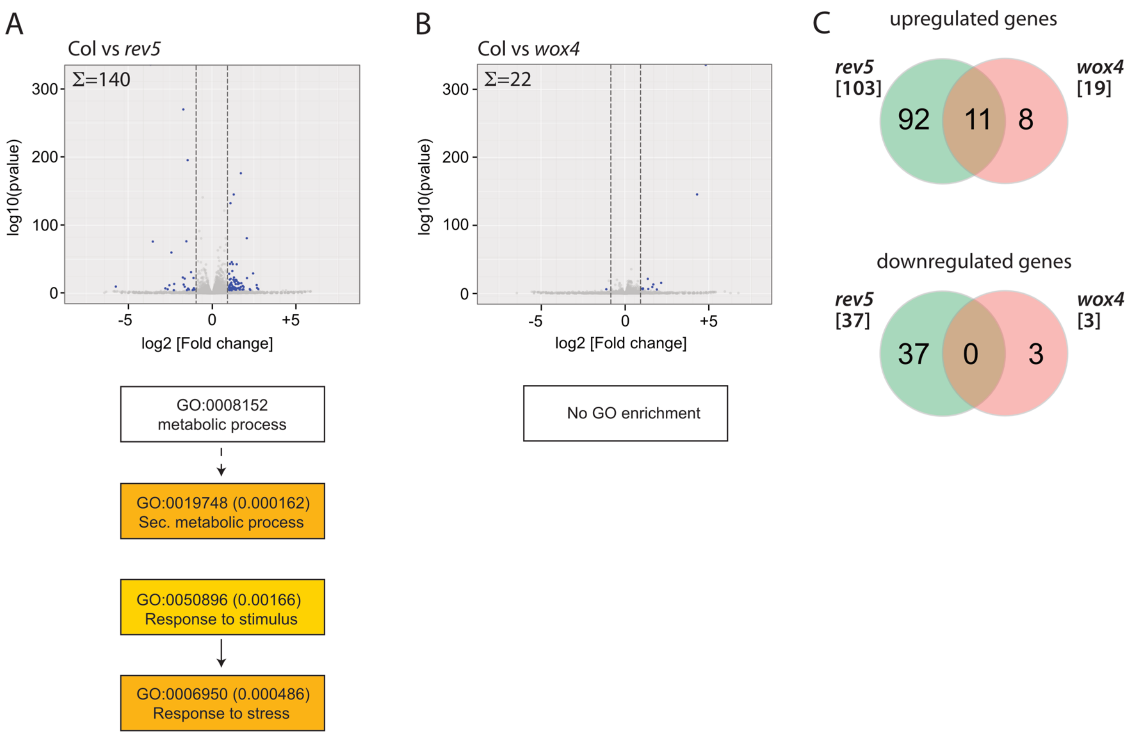

Supplementary Figure S9. Identification of differentially expressed genes in *rev5*, *wox4* and *wox4 rev5* mutant plants.
(A) top: Volcano plot showing differentially expressed genes in *rev5* compared to the Col-0 wild type. Bottom: gene ontology analysis using the agrigo tool showing that genes related to secondary metabolism and stress responses are over-represented.
(B) Volcano plot of *wox4* mutants compared to wild type; no GO enrichment was observed.
(C) Venn diagram showing the overlap of the transcriptome analysis (log2FC > +1/-1; BH-adj p-value <0.001).
