# Supplementary material for: Light affects tissue patterning of the hypocotyl in the shade-avoidance response
Source: PLoS Genet. 2020 Mar 23;16(3):e1008678. doi: 10.1371/journal.pgen.1008678 (PMC7153905; doi:10.1371/journal.pgen.1008678)
Supplement: S10 Fig — A, images of habituated cell culture cells in the non-differentiated state and TE differentiated cells after hormonal induction. Scale bars = 12μm. B, analysis of TE differentiation efficiency in different light qualities. C, determination of the cell density in induced conditions for different light regimes. D, Analysis of spontaneous TE differentiation in different light conditions. E, determination of the cell density of cells grown in different light regimes in the absence of inducing hormones. d = dark, w = white light, fr = white light supplemented with far-red light (n = 4 replicates of 1–2 experiments). (PDF) [file pgen.1008678.s010.pdf]

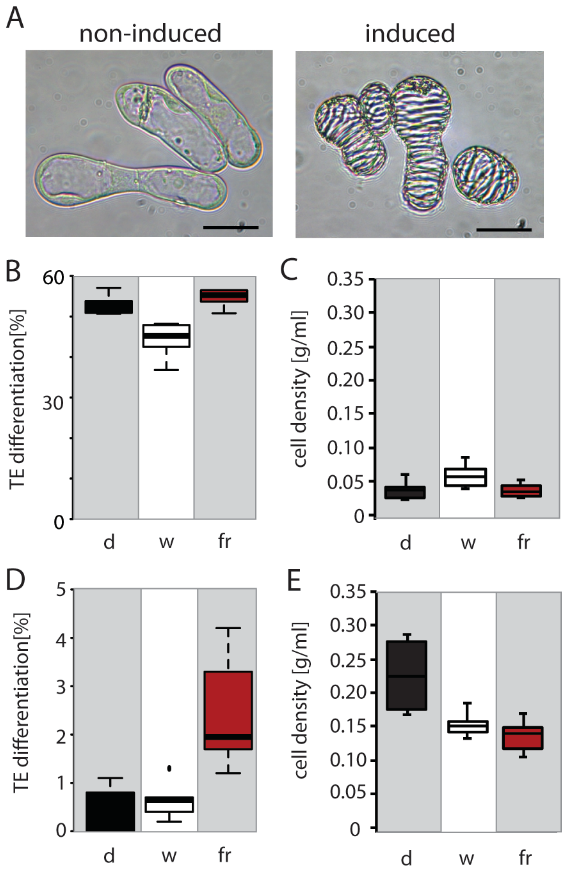

Supplementary Figure S10. Trans-differentiation of TEs *in vitro*.
(A) Images of habituated cell culture cells in the non-differentiated state and TE differentiated cells after hormonal induction. Scale bars = 12µm.
(B) Analysis of TE differentiation efficiency in different light qualities.
(C) Determination of the cell density in induced conditions for different light regimes.
(**D)** Analysis of spontaneous TE differentiation in different light conditions.
(E) Determination of the cell density of cells grown in different light regimes in the absence of inducing hormones. d = dark, w = white light, fr = white light supplemented with far-red light. (n=4 replicates of 1-2 experiments).
