# Supplementary material for: Light affects tissue patterning of the hypocotyl in the shade-avoidance response
Source: PLoS Genet. 2020 Mar 23;16(3):e1008678. doi: 10.1371/journal.pgen.1008678 (PMC7153905; doi:10.1371/journal.pgen.1008678)
Supplement: S11 Fig — Diagram plotting the relative extension of the hypocotyl (y-axis; length of the hypocotyl in WL+BL divided by the length in WL-BL conditions) against the relative changes in TE cell number (x-axis; number of TE cells in WL+BL divided by the number of TE cells in WL-BL conditions) including the added standard errors. (PDF) [file pgen.1008678.s011.pdf]

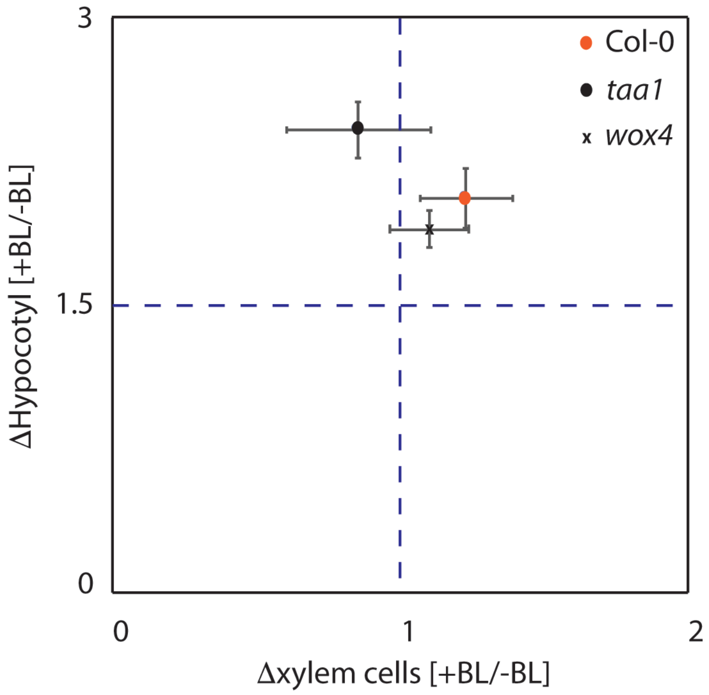


**Supplementary Figure S11. Differential growth analysis of plants grown on medium +/- brassinolide.** Diagram plotting the relative extension of the hypocotyl (y-axis; length of the hypocotyl in WL+BL divided by the length in WL-BL conditions) against the relative changes in TE cell number (x-axis; number of TE cells in WL+BL divided by the number of TE cells in WL-BL conditions) including the added standard errors.
